# DNA Methylation and Protein Markers of Chronic Inflammation and Their Associations With Brain and Cognitive Aging

**DOI:** 10.1212/WNL.0000000000012997

**Published:** 2021-12-07

**Authors:** Eleanor L.S. Conole, Anna J. Stevenson, Susana Muñoz Maniega, Sarah E. Harris, Claire Green, Maria del C. Valdés Hernández, Mathew A. Harris, Mark E. Bastin, Joanna M. Wardlaw, Ian J. Deary, Veronique E. Miron, Heather C. Whalley, Riccardo E. Marioni, Simon R. Cox

**Affiliations:** From the Lothian Birth Cohorts Group, Department of Psychology (E.L.S.C., S.M.M., S.E.H., M.d.C.V.H., M.A.H., J.M.W., I.J.D., R.E.M., S.R.C.), Centre for Genomic and Experimental Medicine, Institute of Genetics and Cancer (E.L.S.C., A.J.S., R.E.M.), Centre for Clinical Brain Sciences (E.L.S.C., S.M.M., M.d.C.V.H., M.E.B., J.M.W., H.C.W.), UK Dementia Research Institute, Edinburgh Medical School (A.J.S., V.E.M.), Division of Psychiatry, Royal Edinburgh Hospital (C.G., M.A.H., H.C.W.), and The Queen's Medical Research Institute, Edinburgh BioQuarter (V.E.M.), University of Edinburgh, UK.

## Abstract

**Background and Objectives:**

To investigate chronic inflammation in relation to cognitive aging by comparison of an epigenetic and serum biomarker of C-reactive protein and their associations with neuroimaging and cognitive outcomes.

**Methods:**

At baseline, participants (n = 521) were cognitively normal, around 73 years of age (mean 72.4, SD 0.716), and had inflammation, vascular risk (cardiovascular disease history, hypertension, diabetes, smoking, alcohol consumption, body mass index), and neuroimaging (structural and diffusion MRI) data available. Baseline inflammatory status was quantified by a traditional measure of peripheral inflammation—serum C-reactive protein (CRP)—and an epigenetic measure (DNA methylation [DNAm] signature of CRP). Linear models were used to examine the inflammation–brain health associations; mediation analyses were performed to interrogate the relationship between chronic inflammation, brain structure, and cognitive functioning.

**Results:**

We demonstrate that DNAm CRP shows significantly (on average 6.4-fold) stronger associations with brain health outcomes than serum CRP. DNAm CRP is associated with total brain volume (β = −0.197, 95% confidence interval [CI] −0.28 to −0.12, *p*_FDR_ = 8.42 × 10^−6^), gray matter volume (β = −0.200, 95% CI −0.28 to −0.12, *p*_FDR_ = 1.66 × 10^−5^), and white matter volume (β = −0.150, 95% CI −0.23 to −0.07, *p*_FDR_ = 0.001) and regional brain atrophy. We also find that DNAm CRP has an inverse association with global and domain-specific (speed, visuospatial, and memory) cognitive functioning and that brain structure partially mediates this CRP–cognitive association (up to 29.7%), dependent on lifestyle and health factors.

**Discussion:**

These results support the hypothesis that chronic inflammation may contribute to neurodegenerative brain changes that underlie differences in cognitive ability in later life and highlight the potential of DNAm proxies for indexing chronic inflammatory status.

**Classification of Evidence:**

This study provides Class II evidence that a DNAm signature of CRP levels is more strongly associated with brain health outcomes than serum CRP levels.

Low-level systemic chronic inflammation has emerged as a hallmark and potential driver for individual differences in brain aging.^1-5^ Yet while chronic inflammation has been consistently linked to dementia,^6-8^ studies investigating peripheral inflammatory markers in nonclinical groups show disparity with respect to cognitive outcomes^9-14^ and have not yet clarified the magnitude and regional extent of brain structural associations.^13-18^

One reason for this inconsistency is that there are no standard biomarkers for chronic inflammation, and to date many studies have relied upon blood biomarkers of acute inflammation such as C-reactive protein (CRP). A significant caveat of this approach is assuming baseline inflammation status from highly phasic protein levels, which are subject to swift and rapid concentration changes in blood plasma.^19^ This introduces significant noise at the epidemiologic level^20,21^ (Figure 1D) and few studies take repeat measures of serum CRP^12^ or attempt to correct for within-person fluctuations. A more accurate reflection may come from an epigenetic approach: DNA methylation (DNAm) profiles have been identified in inflammatory diseases^22,23^ and inflammation-related disease outcomes^24,25^ and are theorized to provide more stable reflections of inflammatory exposure.^24,26-28^ In the same cohort as in the present study, a DNAm proxy of CRP exhibited greater longitudinal stability and stronger associations with cognitive functioning than serum CRP levels.^11,28^

**Figure 1:**
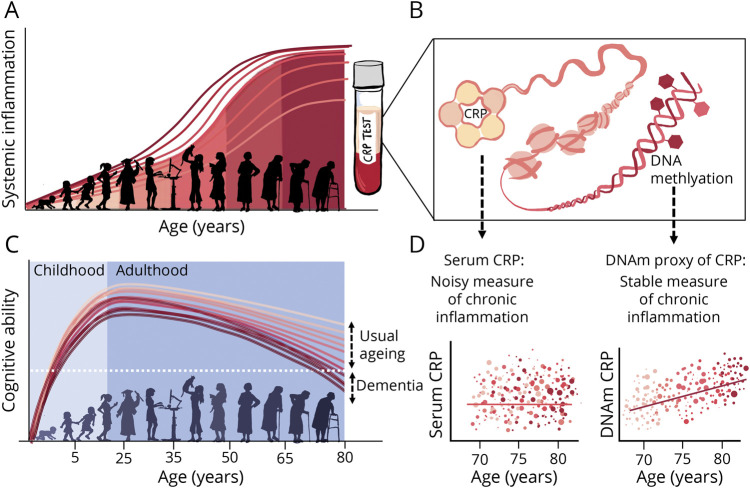
Chronic Inflammation Increases With Age and May Contribute to Variance in Cognitive Ability (A) Schematic demonstrating lifespan curves for chronic inflammation. Inflammatory load tends to increase with age; lifestyle, genetics, and health conditions can all influence susceptibility to chronic inflammation and account for variance in inflammation levels between individuals.^1,3^ (B) Chronic inflammation can be measured by inflammatory proteins taken from a blood sample, such as serum levels and DNA methylation (DNAm) proxies of C-reactive protein (CRP). (C) Lifespan curves for cognitive ability outlining how there is considerable interindividual heterogeneity in rate and timing of cognitive decline, with some people on more accelerated cognitive aging trajectories than others.^4^ (D) Trajectories of LBC1936 participants respective of inflammation scores over age, as outlined in Stevenson et al,^11^ illustrating comparative stability of DNAm inflammation marker compared to serum CRP.

Here, we predict that a DNAm signature of CRP will show significantly stronger associations with brain health outcomes than its serologic counterpart. Our objective is to examine the relationship between chronic inflammation, brain structure, and cognitive aging in a large community-dwelling sample of older adults.

## Methods

### Participants

The Lothian Birth Cohort 1936 (LBC1936) comprises individuals who were surviving members of the Scottish Mental Survey 1947, born in 1936, and who were living in Edinburgh and the surrounding area (the Lothians) when the study began in 2004. Full details of the recruitment procedures and protocols have been published.^29^ Participants took part in 4 waves of testing in later life (at mean ages 70, 73, 76, and 79 years) as part of an investigation into the determinants of cognitive aging. At each wave, participants were interviewed and tested individually by a trained psychologist and a research nurse during a visit to the Wellcome Trust Clinical Research Facility (wtcrf.ed.ac.uk), Western General Hospital, Edinburgh, United Kingdom. This visit included cognitive and other psychological assessments, physical examinations, extensive history taking, and blood analyses. From wave 2 onwards, neuroimaging data are also available.

The current study on chronic inflammation is cross-sectional (all variables described here were collected in 2007, at wave 2 of the LBC1936 study) and addresses the following primary research questions:

1. Does an epigenetic inflammation measure (DNAm CRP) show stronger associations with brain structure and function than serum CRP levels? (Class II evidence)
2. Does an epigenetic inflammation measure (DNAm CRP) show stronger associations with white matter (WM) microstructure than serum CRP levels? (Class II evidence)
3. To what extent can alterations in brain structure explain the association between inflammation and cognitive ability? (Class II evidence)

**Figure 2:**
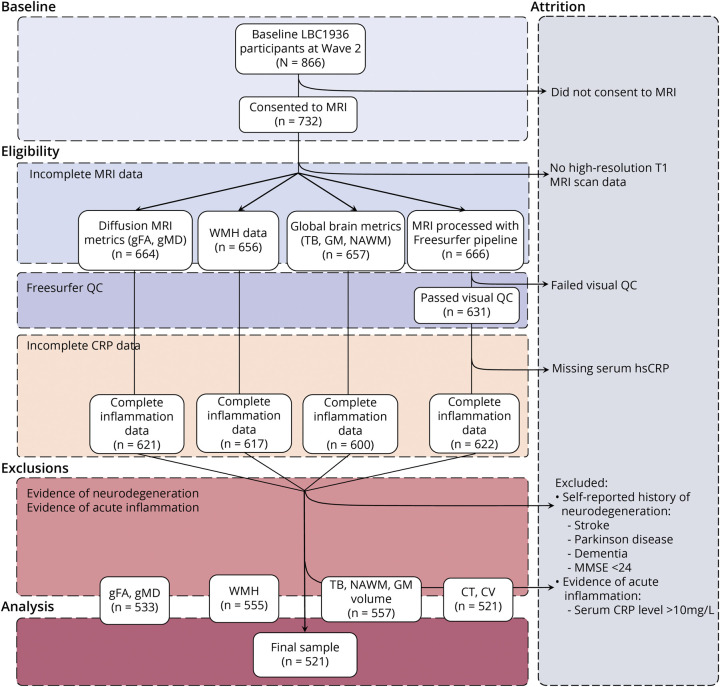
Flowchart Depicting the Step-by-Step Selection Process of the Lothian Birth Cohort 1936 (LBC1936) Participants Included in the Final Sample for Data Analyses CRP = C-reactive protein; CT = cortical thickness; CV = cortical volume; gFA = general fractional anisotropy; GM = gray matter; gMD = general mean diffusivity; hsCRP = high-sensitivity C-reactive protein; MMSE = Mini-Mental State Examination; NAWM = normal appearing white matter; QC = quality control; TB = total brain volume; WMH = white matter hyperintensities.

Participants were free from neurodegenerative diagnoses at baseline and were excluded if they had a self-reported history of stroke, Parkinson disease, or Alzheimer disease or had a Mini-Mental State Examination score <24, indicating mild cognitive impairment. We also excluded participants with serum CRP level >10 mg/L, suggestive of acute infection or illness at the time of blood draw. After exclusions, a total of 521 participants had complete inflammation, cognitive, neuroimaging, and relevant health data. For further details on data availability and attrition, see Figure 2 and eTable 1 (links.lww.com/WNL/B629).

### Brain Imaging Data

Structural and diffusion tensor imaging MRI acquisition and processing in LBC1936 were performed according to an open-access protocol.^29^ A 1.5T GE Signa HDx clinical scanner (General Electric) was used to collect structural T1 (voxel size 1 × 1 × 1.3 mm), T2 (voxel size 1 × 1 × 2 mm), T2* (voxel size 1 × 1 × 2 mm), and fluid-attenuated inversion recovery–weighted images (voxel size 1 × 1 × 4 mm); for full details on MRI sequence measures, refer to Table 1 in the open access protocol article.^29^ Local processing and quality control (QC) of cortical reconstruction and segmentation was performed using FreeSurfer v5.1 on T1-weighted volumes. Full information on brain imaging acquisition, QC, and variables used in analyses is detailed in the supplementary eMethods (links.lww.com/WNL/B629).

**Table 1:**
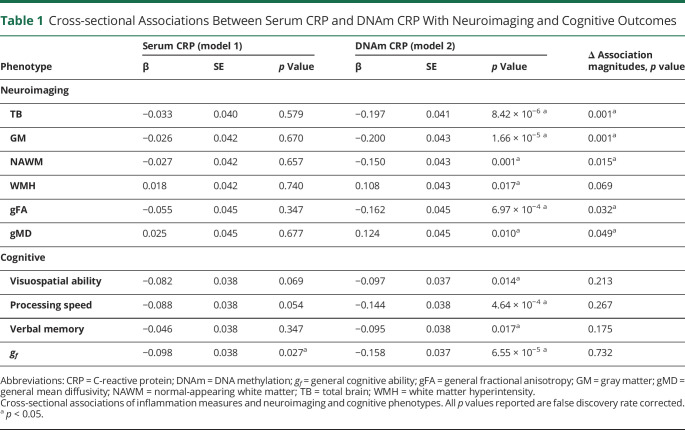
Cross-sectional Associations Between Serum CRP and DNAm CRP With Neuroimaging and Cognitive Outcomes

### CRP Data

Serum CRP was measured from whole blood samples using a high-sensitivity assay (ELISA; R&D Systems).

### DNAm Preparation and DNAm CRP Score

Genome-wide DNAm was measured in blood samples using the Illumina Human MethylationEPIC BeadChip at the Edinburgh Clinical Research Facility Genetics Core; the epigenetic measure of chronic inflammation was calculated for each participant as described previously.^11^ Briefly, a DNAm CRP score was assembled for each participant in wave 2 of LBC1936; this was created by means of a weighted composite score, based on a discovery meta-analysis (9 cohorts, n = 8,863) and a replication meta-analysis (4 cohorts, n = 4,111) of CRP–epigenome-wide association studies (EWAS).^22^ Methylation beta values were derived for the 7 CpG sites shown to have the strongest association with serum CRP levels, then multiplied by their standardized regression weights and added together. Given that all regression weights from the EWAS were negative, a higher DNAm CRP score (i.e., closer to 0) corresponds to a higher inflammatory profile. Relative weights for the 7 CpGs are included in the supplementary document (eTable 2, links.lww.com/WNL/B629).

### Cognitive Ability Data

All participants in the LBC1936 underwent a detailed battery of standardized cognitive tests. From these, participant scores for 3 distinct cognitive domains (visuospatial ability, processing speed, and verbal memory) alongside a general fluid-type cognitive ability score (*g*_*f*_) were created based upon well-fitting, hierarchical structural equation models tested in our previously published work^30^; relevant cognitive tests and individual weightings can be found in the supplementary document (eTable 3, links.lww.com/WNL/B629).

### Lifestyle Variables

Building from previous work that looked at the impact of vascular risk factors (VRFs) on cognitive aging,^31^ we selected the most pertinent variables available to us in the LBC1936 cohort that may influence or confound the relationship between inflammation, brain health, and cognitive aging. Lifestyle variables included body mass index (BMI; kg/m^2^), calculated from height and weight at the time of interview (see eMethods for details), alongside variables relating to self-reported health and disease history: cardiovascular disease history (CVD), hypertension, diabetes, smoking status (coded as current smoker [1] vs ex/nonsmoker [0]), and alcohol use (coded as drinker [1] vs nondrinker [0]). Regular anti-inflammatory drug use was also collected at baseline and coded as on medication [1] or not on medication [0].

### Statistical Analyses

Statistical analyses were performed in R version 3.6.1 (rproject.org). Alpha was 0.05 for all analyses and results were corrected for multiple comparisons using the false discovery rate (FDR).^32^ Standardized coefficients are reported throughout to facilitate comparison of associations. Serum measures of CRP were log-transformed to correct a positively skewed distribution. White matter hyperintensity (WMH) volume was log transformed, after which it showed an approximately normal distribution. All global MRI volumetric measures (total brain [TB], gray matter [GM], normal-appearing WM [NAWM], WMH) were corrected for intracranial volume (ICV) and expressed as a ratio of ICV. For volumetric brain associations, differences between association magnitudes (serum CRP vs DNAm CRP associations) were assessed using the Williams test^33^ for dependent groups with overlapping correlations (cocor.indep.groups.overlaps) as implemented in the “cocor” R package (cran.r-project.org/web/packages/cocor/cocor.pdf). We ensured that models showed acceptably low multicollinearity (variance inflation was ascertained using “vif” in the “car” package in R; cran.r-project.org/web/packages/car/car.pdf). Pairwise bivariate associations were assessed between markers of inflammation, neuroimaging, and lifestyle covariates using Pearson correlation. All models were adjusted for age and sex. Details of individual analyses are as follows.

### Volumetric Brain Associations With Inflammation

Linear regression models were used to identify the proportion of phenotypic variance explained by DNAm CRP and to determine whether this was independent of the serum CRP signal for each brain health phenotype. Logistic regressions were conducted for self-reported disease history variables with binary outcomes (disease/no disease).

### Regional Brain Analyses

Localized associations between DNAm CRP score and vertex-wise cortical volume, area, and thickness were performed using linear regression, controlling for age, sex, and ICV. We used the SurfStat MATLAB toolbox (math.mcgill.ca/keith/surf stat) for Matrix Laboratory R2012a (The MathWorks, Inc.). The resulting statistical maps (*t*-maps) were corrected for multiple comparisons using FDR with a q value of 0.05 across all 327,684 vertices on the cortical surface.

### Sensitivity Analyses

Ancillary mixed-effects models including interaction terms (e.g., inflammation × age, inflammation × sex, inflammation × anti-inflammatory drug use) investigated whether the association of chronic inflammation with brain health outcomes was modified or confounded by age, sex, or the use of anti-inflammatory medication (eTable 4, links.lww.com/WNL/B629). Similarly, we included lifestyle and health covariates in a fully adjusted model (alongside age and sex) to determine whether individual aspects of health and lifestyle had an impact on the association of inflammation with brain health phenotypes (eTable 5, links.lww.com/WNL/B629).

### Mediation Analyses

We ran mediation analyses in a structural equation modeling (SEM) framework using the R “lavaan” package (cran.r-project.org/web/packages/lavaan/lavaan.pdf). This simultaneously characterized associations among CRP, brain, and cognitive metrics, and also specifically tested the hypothesis that brain structure would partly and significantly mediate associations between measures of CRP and cognitive ability. Both single and multiple mediator models were specified (see Figure 5, A–C, as example). Single mediator models provided information on the proportion of CRP–cognitive associations attributable to individual neuroimaging metrics. By contrast, in multiple mediator models, brain structural variables were entered simultaneously as covarying mediators (see path diagram, Figure 5C). This allowed us to quantify the proportion of variance in CRP–cognitive associations uniquely explained by each facet of brain structure (GM, NAWM, WMH, general fractional anisotropy [gFA], general mean diffusivity [gMD]). The primary estimates of interest in this study are the degree of change (mediation) in the direct path (c to c′) between inflammation measures (DNAm CRP or serum CRP) and cognitive ability (*g*_*f*_ or processing speed or visuospatial ability or verbal memory) when the indirect path from inflammation to cognitive ability via brain structure (a × b) is included. A significant mediation of the c path (to c′) is denoted by the statistical significance of this indirect effect. Bootstrapping was used to calculate standard errors. Multiple comparisons were corrected for by FDR correction. These mediations were re-run when accounting for self-reported health variables as covariates: in model 1, age and sex were covariates; in model 2, they were age, sex, BMI, hypertension, diabetes, smoking status, and alcohol use. To account for missing data bias, we took account of all available data, using full information maximum likelihood estimation.^34^ Model fit was evaluated based on root mean squared error approximation (RMSEA), the comparative fit index (CFI), the standardized root mean square residual (SRMR), and the Tucker–Lewis index (TLI). We considered a model an acceptable fit when it respected the following thresholds: RMSEA ≤0.05; SRMR ≤0.06; CFI ≥0.97; and TLI ≥0.95, as recommended.^35^

### Data Availability

The data analyzed in this study are not publicly available as it contains data that could compromise participant consent and confidentiality, but can be requested via a data access request to the Lothian Birth Cohorts research group.

### Standard Protocol Approvals, Registrations, and Patient Consents

Ethical permission for the LBC1936 was obtained from the Multi-Centre Research Ethics Committee for Scotland (MREC/01/0/56) and the Lothian Research Ethics Committee (LREC/2003/2/29). Written informed consent was obtained from all participants. All necessary patient/participant consent has been obtained and the appropriate institutional forms have been archived.

## Results

### DNAm CRP Is Associated With Global and Regional Brain Volume

We studied 521 eligible older adults (aged ∼73 years; refer to Figure 2 and eTable 1, links.lww.com/WNL/B629) and looked at epigenetic vs serum inflammation associations across a range of neuroimaging and cognitive measures (Table 1). To index chronic inflammation, an epigenetic measure of CRP (DNAm CRP) was assembled for each participant (see Methods). The correlation between the DNAm CRP score and serum log(CRP) was moderate (*r* = 0.29, 95% confidence interval [CI] 0.28–0.4), and the DNAm CRP score showed a stronger correlation with serum CRP than any one of its composite CpGs.^11^

We found that higher inflammatory burden, indexed by DNAm CRP scores, associated with poor cognitive and neuroimaging brain health outcomes (Table 1). DNAm CRP exhibited significantly larger (6.4-fold, on average) associations with brain structural MRI metrics (including global GM and WM atrophy, poorer WM microstructure, and increased WMH burden) than serum CRP. These DNAm CRP-associated brain structural changes were independent of anti-inflammatory drug use, age, or sex (eTable 4, links.lww.com/WNL/B629). Participants with a higher inflammatory burden on average had greater overall brain atrophy, with higher DNAm CRP associating with lower total brain volume (β = −0.197, 95% CI −0.28 to −0.12, *p*_FDR_ = 8.42 × 10^−6^), GM volume (β = −0.200, 95% CI −0.28 to −0.12, *p*_FDR_ = 1.66 × 10^−5^), and WM volume (β = −0.150, 95% CI −0.23 to −0.07, *p*_FDR_ = 0.001). Models that included additional health and lifestyle covariates (BMI, smoking, alcohol consumption, hypertension, diabetes, and cardiovascular disease history) attenuated the relationship between DNAm CRP and brain health outcomes by up to 40% (eTable 5, links.lww.com/WNL/B629). Of these, the associations between DNAm CRP with WM measures (WMH, NAWM) were the most attenuated (34%–40%). Out of the lifestyle and health factors accounted for, smoking appeared to have the greatest influence on the attenuation (as illustrated in supplementary eFigure 2, links.lww.com/WNL/B629).

**Figure 3:**
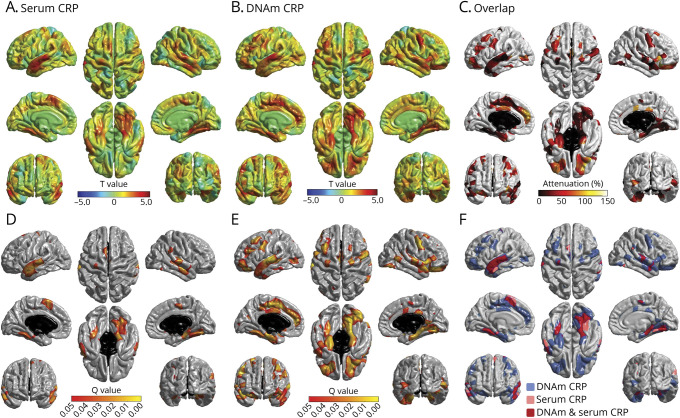
DNAm CRP Shows Stronger and More Widespread Associations With Global and Regional Brain Structure Than Serum CRP Regional cortical volume regressed against serum C-reactive protein (CRP) (A, D) and DNA methylation (DNAm) CRP (B, E) (n = 521). Colors denote the magnitude (T-maps; top) and significance (Q values; bottom) of the negative associations between inflammation and brain cortical volume. (C) Percentage attenuation for the significant associations between DNAm-CRP and cortical volume when also controlling for serum CRP. Conjunction plot (F) shows the spatial extent of independent contributions and overlap (red) in cortical loci that exhibit false discovery rate (FDR)–corrected unique associations with simultaneously modeled serum (pink) and epigenetic (blue) inflammation measures; results are corrected for sex, age, and intracranial volume.

After examining global brain structural alterations, we looked at specific regional cortical brain associations with higher inflammation levels. We found regional heterogeneity in the patterning of associations between CRP measures and cortical metrics: atrophy in frontal, anterior lateral, and medial temporal lobes was associated with higher DNAm CRP (Figure 3B); inflammation associations with brain cortical thickness are presented in the supplementary document (eFigure 1, links.lww.com/WNL/B629). Overall, these results emphasize that the DNAm-CRP score associates with lower cortical volume of specific brain regions (lateral and medial temporal regions of the brain), which show overlap with those of serum CRP and unique variance (Figure 3F), with DNAm CRP reflecting atrophy beyond the serum CRP score.

### DNAm CRP Is Associated With White Matter Microstructure in Specific White Matter Tracts

**Figure 4:**
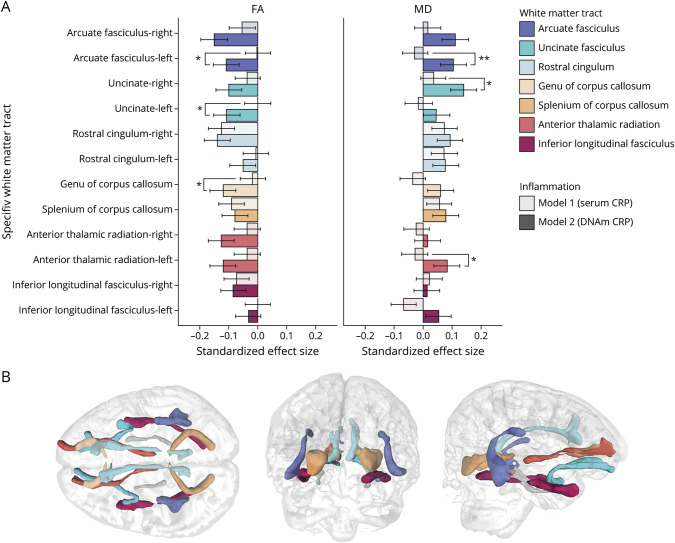
DNA Methylation C-Reactive Protein is Associated With White Matter Microstructure in Specific White Matter Tracts (A) Standardized regression coefficients for associations between white matter tract–averaged fractional anisotropy (FA; left) and mean diffusivity (MD; right). Bars show standardized coefficients and standard errors. Asterisks indicate where associations are significantly larger for DNA methylation than for serum (*p < 0.05, **p < 0.01). (B) Illustration of the respective white matter tracts measured using probabilistic neighborhood tractography in one LBC1936 study participant.

Next, we investigated whether higher DNAm CRP was related to lower WM microstructure based on global and regional diffusion MRI (dMRI) measures by looking at inflammation associations with WM tract fractional anisotropy and mean diffusivity. Whereas serum CRP–dMRI associations were null in all cases (all *p*_FDR_ >0.089) (eTables 6–7, links.lww.com/WNL/B629), higher DNAm CRP scores predicted overall lower gFA (β = −0.162, *p*_FDR_ = 6.94 × 10^−4^) and higher gMD (β = 0.124, *p*_FDR_ = 0.010). For specific WM tracts, the strongest associations were seen for the arcuate fasciculus and uncinate fasciculus, with lower FA and higher MD with higher DNAm CRP (see Figure 4; eTables 6–7, links.lww.com/WNL/B629). For global measures of WM tract integrity (gFA, gMD), accounting for health and lifestyle covariates did not substantially alter the magnitude or significance of these associations (eTable 5, links.lww.com/WNL/B629); however, at the level of individual WM tracts, the relationship between DNAm CRP and FA and MD was attenuated when lifestyle factors were included in the models (eTables 8–9, links.lww.com/WNL/B629); this is illustrated in supplementary eFigure 3 (links.lww.com/WNL/B629).

### Brain Structure Partly Mediates the Association of DNAm CRP With Cognitive Ability

**Figure 5:**
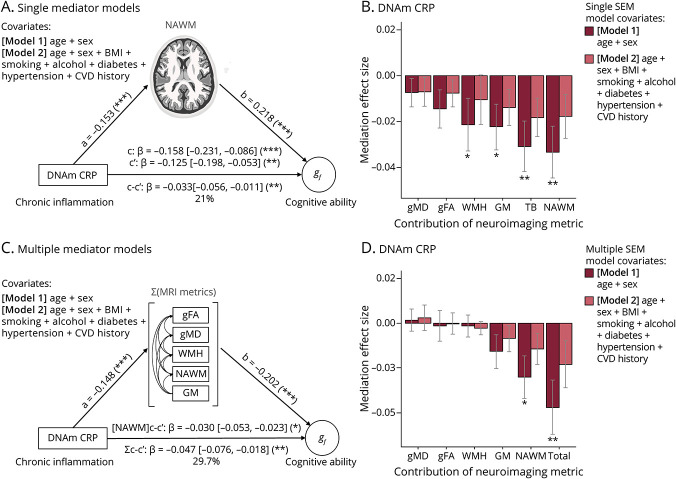
Brain Structure Partly Mediates the Association of DNAm CRP With Cognitive Ability Top panel (A, B) displays single mediator models, bottom panel (C, D) displays multiple mediator models. (A) Model 1 structural equation model path diagrams showing that in model 1, the association between DNA methylation (DNAm) C-reactive protein (CRP) and general cognitive ability (path c) was significantly and partially mediated by normal-appearing white matter volume (path ab = −0.033, *p* = 0.001), attenuating the c path by 21% (path c′) and (C) 29.7% by multiple MRI variables (ab = −0.047, *p* = 0.002). (B) Single mediator models indirect effect size and standard error bars. (D) Multiple mediator models indirect effect size and standard error bars. Light bars show model 1 (includes covariates age and sex), dark bars show model 2, which contains additional health covariates (age + sex + body mass index [BMI] + hypertension + smoking status + alcohol use + cardiovascular disease [CVD] history + diabetes); *p < 0.05; **p < 0.01. *g*_*f*_ = general cognitive ability; gFA = general fractional anisotropy; GM = gray matter; gMD = general mean diffusivity; NAWM = normal-appearing white matter; WMH = white matter hyperintensity.

As higher DNAm CRP levels were associated with lower cognitive performance both here (Table 1) and previously,^11^ we quantified the degree to which brain structural differences contribute to the inflammation–cognition association, and which facets show the strongest unique contributions to this relationship using an SEM framework. Bivariate associations between all variables (inflammation, brain structure, cognitive ability, and lifestyle measures) are provided in eTable 10 (links.lww.com/WNL/B629). Whereas TB volume, GM volume, NAWM volume, and WMH volume all emerged as significant mediators in single SEM models (percentage attenuation 14%–21%; eTable 11, links.lww.com/WNL/B629), multiple mediator models were used to test the degree to which each global MRI metric contributed uniquely to mediation of the same association (Figure 5D; eTable 12, links.lww.com/WNL/B629). Here, the sum total of MRI measures significantly mediated the association between DNAm CRP and general cognitive ability (β = −0.047 [−0.076 to −0.018], *p*_FDR_ = 0.002; percentage attenuation 29.7%). The unique contributions to this variance were largest for NAWM volume (β = −0.03 [−0.053 to −0.023], *p*_FDR_ = 0.012), indicating that the loss of WM may contribute to inflammation-associated differences in cognitive functioning in older age. Out of the individual cognitive domains, processing speed was the most significantly mediated by the sum total of MRI metrics (β = −0.058 [−0.090 to −0.027], *p*_FDR_ = 0.001; percentage attenuation 41%). Again, NAWM emerged as the largest unique contribution to this variance (β = −0.037 [−0.053, −0.007], *p*_FDR_ = 0.006; eTable 13 [links.lww.com/WNL/B629]). Similarly, visuospatial ability was significantly mediated by the sum total of MRI metrics (β = −0.036 [−0.063 to −0.010], *p*_FDR_ = 0.013; percentage attenuation 37%), with NAWM accounting for the largest unique contribution to this effect (β = −0.030 [−0.063 to −0.010], *p*_FDR_ = 0.012). While verbal memory was significantly mediated by the sum total of MRI metrics (β = −0.031 [−0.057 to −0.007], *p*_FDR_ = 0.026; percentage attenuation 33%), there were no significant contributions from individual MRI metrics (eTables 12–13, links.lww.com/WNL/B629).

Finally, with the addition of lifestyle and health covariates to our models, no aspect of brain structure remained a significant mediator of the associations between DNAm CRP and general cognitive ability (β_mediation_ = −0.023 [−0.049 to 0.003], *p*_FDR_ = 0.167 (see Figure 5, eTables 11–12, links.lww.com/WNL/B629) or any of the individual cognitive domains (eTable 12, links.lww.com/WNL/B629).

## Discussion

Only recently has there been a push for integrated multi-omics approaches to better characterize chronic inflammation.^3,26^ DNAm profiles may act as promising peripheral biomarkers for cognitive–aging differences at the population level, given their relative stability in the short term, and their joint modulation by both genetic and lifestyle traits. Elsewhere, DNAm markers of inflammation have proved informative in predicting a range of age-related health outcomes, from cardiovascular disease to depression,^23,24,36^ but few studies have applied this same approach to cognitive aging differences in healthy cohorts. As chronic inflammation is considered to be an insidious, cumulative, and often undetected contributor to cognitive aging,^1,3,5,14^ the importance of such epigenetic markers may be their utility to index inflammatory load with greater reliability than phasic protein measures. In this study, DNAm CRP was more robustly associated with a range of cognitive and neuroimaging metrics than serum CRP, supporting our original hypothesis. We discovered that DNAm CRP shows consistently stronger associations with brain structure than serum CRP (on average, 6.4-fold greater), that these associations are not regionally homogeneous across the brain's cortex, and that specific aspects of brain structure partly mediate (up to 29.7%) associations between an epigenetic signature of CRP and cognitive functioning. Our results highlight the potential of epigenetic approaches to indexing inflammation in population cohorts and suggest that chronic inflammation may contribute to both focal and global brain structural changes that underlie differences in cognitive aging.

We found regional heterogeneity in the patterning of associations between CRP measures and cortical metrics, indicating differential regional vulnerability to chronic inflammation.

Reductions in brain cortical volume and thickness in frontal, anterior lateral, and medial temporal lobes were associated with increased DNAm CRP. Consistently, previous studies report structural changes associated with inflammatory markers in the temporal and frontal cortices.^17,18^ Atrophy in these regions is implicated in cognitive decline,^37^ and differential patterns of proinflammatory receptor distribution may underlie why some brain regions are more vulnerable to inflammation than others. For example, in patients with Alzheimer disease (AD), proinflammatory cytokine receptor density and expression are increased in regions of neurodegeneration, including the medial frontal and temporal cortices.^38^ Higher inflammation levels have also been related to progression of atherosclerosis, with evidence for differential effects of CRP in different beds of the arterial brain supply.^39,40^ These findings suggest that raised levels of inflammatory mediators may contribute to localized brain atrophy via their differential expression in brain tissue and cerebrovasculature.

Overall, the results of our mediation analyses indicate that chronic inflammation's detrimental effect on WM beyond other brain structural features may underlie the inflammation-associated differences in cognitive functioning in older age. Whereas numerous studies have found associations between reduced WM volume and raised inflammatory markers in healthy cohorts^14,17,41^ and those with chronic inflammatory conditions,^42^ few have looked at inflammation, brain structure, and cognitive function concurrently.^13^ Fewer still have attempted to more robustly characterize chronic inflammation beyond assessing serum inflammatory protein profiles—although a notable exception comes from recent work, where the same DNAm CRP signature was found to be significantly associated with widespread reductions in WM integrity beyond that of serum CRP.^36^ In agreement with this study, but in an older cohort, we found that higher DNAm CRP related to increased WMH burden, reduced NAWM volume, and ostensibly poorer WM microstructure (lower FA and higher MD). In particular, the WM tracts of arcuate fasciculus and uncinate fasciculus showed the most consistent significant relationships with DNAm CRP levels (across both FA and MD), alongside significantly lower FA in the anterior thalamic radiation, which are consistent with studies assessing the effects of vascular risk on microstructure with advanced age.^31^

**Figure 6:**
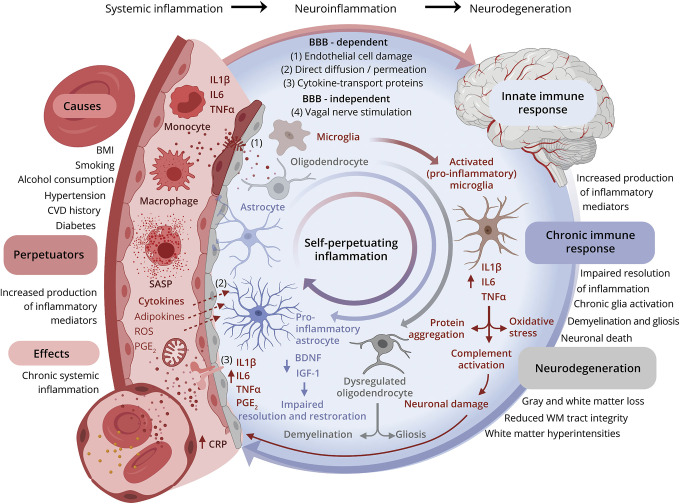
Mechanisms of Neurodegeneration via Increased Systemic Chronic Inflammation (A) Chronic inflammation is pertinent to brain aging in that inflammatory mediators in the periphery can damage the blood–brain barrier (BBB), permitting entry into the brain where they go on to disrupt neurons and glia and perpetuate a chronic inflammatory state. This directly contributes to various neurodegenerative pathways (illustrated) that lead to brain cell death. (B) Suggested mechanisms by which the causes of inflammaging (immunosenescence, lifestyle, clinical health) and related consequences may drive brain health (structural and cognitive) outcomes. (C) Study model: chronic inflammation is a key driver of cognitive decline through its effects on brain structure. Left shows generic directed acyclic graph for mediation analysis (left panel) and for the study example (right panel). A = exposure; BDNF = brain-derived neurotrophic factor; C = confounder; CRP = C-reactive protein; IGF-1 = insulin-like growth factor 1; IL1 β = interleukin-1β; IL6 = interleukin-6; M = mediator; PGE2 = prostaglandin E2; ROS = reactive oxygen species; SASP = senescence associated secretory phenotype; TNF-α = tumor necrosis factor-α; Y = outcome. Created with BioRender.com.

In agreement with longstanding findings from neurocognitive studies—where, consistently, inflammation is more strongly associated with declines in processing speed than other cognitive domains^9,10^—our results indicate that some cognitive domains (processing speed) may be more mediated by the brain structural consequences of chronic inflammation than others (verbal memory, visuospatial ability). Processing speed has been strongly linked to WM integrity at both global and regional levels^43^ and many of the downstream effects of neuroinflammatory processes directly affect WM integrity (to include demyelination, de-afferentation and gliosis; see Figure 6 and eFigure5, links.lww.com/WNL/B629).^14^ As such, chronic inflammation's contribution to diffuse and global WM loss may disproportionately affect cognitive functions that require the coordination of brain regions (e.g., processing speed), compared to more functionally localized ones (e.g., verbal memory). However, we have not formally compared the magnitude of these attenuations, and judge that this greater degree of attenuation is likely to be a general shared process^30^ plus some degree of noise, given that variance across cognitive domains is shared at the general level.^30,44^

The attenuation seen in inflammation–brain health associations when lifestyle factors were accounted for is to be expected given what is known about inflammation and VRFs on brain–health outcomes.^3,31^ Vascular inflammation is considered to be a shared mechanism linking cardiometabolic factors (to include hypertension and smoking) with poor cognitive outcomes.^31,40,45^ Figure 6 models this relationship and illustrates how increased inflammation in the periphery can result in neurodegenerative processes via BBB-dependent and independent pathways.^46^ The regional areas of brain loss that were particularly associated with DNAm CRP are also areas where others have shown increased BBB leakage in persons at risk of AD.^47^ Peripheral markers of inflammation such as CRP have been consistently linked to the risk of cerebrovascular and cardiovascular events,^1,3,5,40^ and there is evidence that atherosclerotic and thrombotic presentations signify a chronic inflammatory process.^20,39^ The methylation CRP score was more strongly associated with modeled VRFs (hypertension, CVD history, diabetes, alcohol consumption, and smoking) than serum CRP (eTable 10, links.lww.com/WNL/B629). Given that the 7 CpGs that make up the DNAm CRP score reside in inflammation and vascular-related genes (see eTable 2, links.lww.com/WNL/B629), these DNAm CRP–brain MRI associations may be capturing the effect of upstream inflammatory activity beyond that of serum CRP levels, which may explain why some VRFs show greater association with the DNAm score. Upstream inflammatory cytokines such as interleukin-6 and tumor necrosis factor–α have been shown to be associated with increased risk of dementia where serum CRP levels showed no association, such as in the Rotterdam Study,^6^ Whitehall II longitudinal cohort study,^12^ and Framingham Study.^8^ Variation and measurement error in serum CRP levels may be confounding the relationship between chronic inflammation and related health and lifestyle triggers and exacerbators.^20,21^ Our results add to the evidence base that DNAm-based predictors of inflammation may act as a quantifiable archive of the longitudinal effects of these exposures^11,27,36^ and other unaccounted for health and genetic profiles that serum CRP levels fail to capture.

Strengths of this study include the large sample size, array of multimodal data, and that inflammation, lifestyle, cognitive, and structural brain variables were measured in the same individuals at about the same time. Compared to recent EWAS–neuroimaging research, this study is exceptionally well-powered with 521 participants after exclusions.^48^ Alongside the narrow age range, the homogeneity of the LBC1936 cohort (e.g., all participants are of Scottish ancestry) may have minimized any potentially strong confounding effects that factors such as mixed ethnicity and geography might have had in a more heterogenous sample. This cross-sectional nature means we are unlikely to capture the effect of more age-related changes in inflammatory profile and cognitive decline. Although we endeavored to remove participants with cognition-related pathology, these were screened via self-reported diagnoses, and we may be missing undiagnosed or subclinical incident neurodegenerative pathology. Similarly, while we have identified a range of health and lifestyle variables that could influence inflammatory load (BMI, diabetes, CVD history, smoking, alcohol consumption, hypertension), there are many nonmodeled variables that could contribute to this effect, as discussed in depth elswhere.^1,3^

Finally, a clear limitation of the study is that our epigenetic surrogate of inflammation was measured in blood rather than brain tissue. While brain-based biomarkers are the optimal choice for investigating cognitive outcomes, it is impractical to profile such methylomes in brain tissue in living humans. Furthermore, the use of postmortem brain tissue samples has its own problems (in particular, the stability of global DNAm following death^49^) and cannot reliably reflect the plastic state of methylomes in vivo. Future studies should consider examining a wider range of DNAm inflammatory markers (DNAm levels of interleukins, prostaglandins, and neurotrophins); DNAm inflammatory markers in younger participants (where there is likely greater variation in baseline inflammation levels); DNAm inflammatory markers in specific brain pathology cases (e.g., multiple sclerosis); as well as how peripheral inflammatory and neuroinflammatory DNAm patterns equate, and how each relates to cellular differences within the brain to give rise to the structural alterations we observe here.^50^

Our findings do not establish causality but support the hypothesis that chronic systemic inflammation may contribute to neurodegenerative brain changes that underlie differences in cognitive ability in later life. Previous studies exploring this relationship may underestimate the brain and cognitive sequelae of chronic inflammation by relying on single measurements of phasic serum proteins. By using an epigenetic inflammation measure, which integrates information from multiple immune-related CpG sites, we may provide a more reliable measure of chronic inflammation and thus a more comprehensive overview of the consequences of chronic inflammation on brain structure and function. Reliable monitoring of inflammatory exposure could enable clinicians to review the efficacy of drug and lifestyle interventions to attenuate inflammation levels with a view to improving cognitive outcomes.

## Glossary

AD: Alzheimer disease
BMI: body mass index
CFI: comparative fit index
CI: confidence interval
CRP: C-reactive protein
CVD: cardiovascular disease
dMRI: diffusion MRI
DNAm: DNA methylation
EWAS: epigenome-wide association studies
FDR: false discovery rate
gf: general cognitive ability
gFA: general fractional anisotropy
GM: gray matter
gMD: general mean diffusivity
ICV: intracranial volume
NAWM: normal-appearing white matter
QC: quality control
RMSEA: root mean squared error approximation
SRMR: standardized root mean square residual
TB: total brain
TLI: Tucker–Lewis index
VRF: vascular risk factor
WM: white matter
WMH: white matter hyperintensity
